# Isolated Splenic Infarcts as a Presentation of Diffuse Large B-Cell Lymphoma

**DOI:** 10.7759/cureus.95465

**Published:** 2025-10-26

**Authors:** Joshua Shadrach Daniel, Noraidah Masir, Wai Yee Chan, Teck Huat Wong, Jay Suriar Rajasuriar

**Affiliations:** 1 Internal Medicine, International Medical University, Kuala Lumpur, MYS; 2 Pathology, Premier Integrated Labs, Kuala Lumpur, MYS; 3 Radiology, Gleneagles Hospital Kuala Lumpur, Kuala Lumpur, MYS; 4 Department of Nuclear Medicine, Pantai Hospital, Kuala Lumpur, MYS; 5 Haematology, Gleneagles Hospital Kuala Lumpur, Kuala Lumpur, MYS

**Keywords:** diffuse large b-cell lymphoma, isolated painful splenic infarcts, myc/bcl-2 dual expression, non-germinal centre b-cell, non-hodgkin lymphoma

## Abstract

Isolated splenic infarcts are a rare presentation of diffuse large B-cell lymphoma (DLBCL). We report the case of a 51-year-old East Asian male who presented with pleuritic chest pain, low-grade fever, and weight loss. Imaging studies revealed progressive splenomegaly with multiple splenic infarcts, while a positron emission tomography-computed tomography (PET/CT) demonstrated hypermetabolic splenomegaly along with small lymph nodes. A lymph node biopsy confirmed DLBCL, non-germinal centre subtype with dual BCL2/CMYC expression and a high Ki67 index. He was treated with R-CHOEP (rituximab, cyclophosphamide, doxorubicin, vincristine, prednisolone, and etoposide), which was later switched to R-CHOP (rituximab, cyclophosphamide, hydroxydaunorubicin, vincristine, and prednisone) due to treatment-related neutropenia. End of treatment PET/CT showed persistent splenic uptake, prompting an elective splenectomy. The surgery was successful and there was no residual disease observed. This case highlights the diagnostic challenges of DLBCL presenting with isolated splenic infarction, emphasizing the role of PET/CT and biopsy in evaluation.

## Introduction

Diffuse large B-cell lymphoma (DLBCL) is the most aggressive and common subtype of non-Hodgkin lymphoma (NHL), representing approximately 20-50% of all NHLs globally. Originating from mature B-cells, DLBCL is characterised by rapid tumour growth and a wide range of clinical presentations [1]. Typically, DLBCL presents with nodal (lymphadenopathy) and extra-nodal (organomegaly) disease, frequently affecting the spleen, liver, skin, bones, and brain. However, isolated splenomegaly causing splenic infarcts is exceedingly rare, occurring in fewer than 1% of patients with non-Hodgkin lymphoma [1,2]. Splenic infarcts are also typically associated with infections, vasculitides, embolic events, and sickle cell disease. In lymphomas, splenic infarcts are attributed to lymphomatous infiltration, which can cause vascular compromise. Here, we present a patient who presented with isolated painful splenic infarcts due to lymphomatous splenic involvement. DLBCL (non-germinal center B-cell (GCB) subtype with MYC/BCL-2 dual expression) was diagnosed via immuno-histochemical and histopathological analysis of a superficial right submandibular lymph node.

## Case presentation

A 51-year-old East Asian male presented to his general practitioner with left lower pleuritic chest and low-grade fever for one week. The chest pain intensified with coughing, deep breathing, and after large meals. The pain was not exertional or radiating to other sites. He attributed the 4 kg weight loss to eating smaller meals. His past medical history was significant for a childhood diagnosis of tuberculosis (TB) of the right 12th rib that had been treated surgically and pharmacologically. He had recently travelled to a rural area and was also an avid hiker. Abdominal examination was grossly normal with no evidence of hepatosplenomegaly.

A chest X-ray was normal, while a non-enhanced computed tomography (CT) scan of the abdomen showed splenomegaly of 16 cm length with the presence of minimal superior pole subcapsular fluid. A comprehensive laboratory testing seen on Table 1 was performed to obtain a complete picture of the patient's illness, including a complete blood count, white blood cell with differentials, liver function tests, urinalysis, and renal profile, which were all unremarkable, except for a mildly raised erythrocyte sedimentation rate of 41 mm/H and an elevated C-reactive protein of 36.4 mg/L. Microscopy of peripheral blood was normal and negative for malaria parasites.

**Table 1:** Summary of laboratory investigations performed upon initial presentation. A complete blood count, white blood cell with differentials, liver function tests, urinalysis, and renal profile were all unremarkable, except for a mildly raised monocyte count of 11.6%, erythrocyte sedimentation rate of 41 mm/H, and an elevated C-reactive protein of 36.4 mg/L. Microscopy of peripheral blood smear was negative for malaria parasites.

Category	Test	Result	Reference Range	Units
Complete Blood Count (CBC)	Red Blood Cells (RBC)	4.85	4.2–5.9	×10⁶/µL
	Red Cell Distribution Width (RDW)	12.8	11.5–14.5	%
	Packed Cell Volume (PCV)	43	38–50	%
	Mean Cell Volume (MCV)	88	80–100	fL
	Mean Cell Hemoglobin (MCH)	30	27–33	pg
	Mean Corpuscular Hb Conc. (MCHC)	34	32–36	g/dL
	White Blood Cells (WBC)	6.3	4.0–11.0	×10³/µL
	Platelets	207	150–450	×10³/µL
WBC Differential	Neutrophils (%)	65.1	40–70	%
	Neutrophils (Abs)	4.1	1.8–7.7	×10³/µL
	Lymphocytes (%)	21.2	20–45	%
	Lymphocytes (Abs)	1.3	1.0–4.8	×10³/µL
	Monocytes (%)	11.6	2–10	%
	Monocytes (Abs)	0.7	0.2–1.0	×10³/µL
	Eosinophils (%)	1.6	1–6	%
	Eosinophils (Abs)	0.10	0–0.5	×10³/µL
	Basophils (%)	0.5	0–1	%
	Basophils (Abs)	0.0	0–0.2	×10³/µL
Liver Function Tests (LFTs)	Total Protein	70	64–83	g/L
	Albumin	44	35–50	g/L
	Globulin	26	20–35	g/L
	Albumin/Globulin Ratio	1.69	1.0–2.1	–
	Alkaline Phosphatase (ALP)	83	40–129	U/L
	Aspartate Transaminase (AST)	25	10–40	U/L
	Alanine Transaminase (ALT)	40	7–56	U/L
	Gamma-Glutamyl Transferase (GGT)	45	10–60	U/L
	Total Bilirubin	11.0	3–17	µmol/L
Renal Function Profile	Sodium (Na)	142	135–145	mmol/L
	Potassium (K)	3.7	3.5–5.1	mmol/L
	Chloride (Cl)	106	98–107	mmol/L
	Urea Nitrogen (BUN)	6.2	2.5–7.1	mmol/L
	Creatinine	85	62–106	µmol/L
	eGFR (CKD-EPI)	>90	≥90	mL/min/1.73m²
	Uric Acid	0.54	0.21–0.43	mmol/L
	Glucose (fasting)	4.3	3.9–5.6	mmol/L
Inflammatory Markers	C-Reactive Protein (CRP)	33.3	<10	mg/L
	Erythrocyte Sedimentation Rate (ESR)	27	0–20	mm/hr
Urinalysis (FEME)	Appearance	Clear	Clear	–
	Colour	Yellow	Yellow	–
	pH	6.0	5.0–8.0	–
	Specific Gravity	1.044	1.005–1.030	–
Urinalysis (FEME)	Appearance	Clear	Clear	–
	Colour	Yellow	Yellow	–
	pH	6.0	5.0–8.0	–
	Specific Gravity	1.044	1.005–1.030	–
	Nitrite	Negative	Negative	–
	Protein	Negative	Negative	–
	Glucose	Negative	Negative	–
	Ketones	Negative	Negative	–
	Urobilinogen	16.0	<17	µmol/L
	Bilirubin	Negative	Negative	–
	Blood	Negative	Negative	–
	Leucocytes	Negative	Negative	–
	RBC (microscopy)	Nil	Nil	–
	WBC (microscopy)	Nil	Nil	–
	Epithelial Cells	Nil	Occasional	–
	Crystals	Nil	Nil	–
	Casts	Nil	Nil	–
	Bacteria	Nil	Nil	–
Other Tests	Malaria Parasite (blood film)	Not Seen	Not Seen	–

Based on these findings, the patient was presumptively treated with a non-steroidal anti-inflammatory drug (NSAID) for a suspected viral infection with reactive splenomegaly and asked to return two weeks later for a follow-up ultrasound. However, 10 days later, the patient returned with worsening left-sided pleuritic chest pain with persistent fever and was hospitalized for further evaluation. Physical examination was significant only for percussion dullness in Traube's space, suggesting splenomegaly. Peripheral lymph nodes and liver were not palpable. Serial blood cultures were negative, and cardiac trans-thoracic echocardiography did not demonstrate features of infective endocarditis. To investigate the cause of splenic infarcts and the vague symptoms the patient displayed, an autoimmune panel was carried out. Autoimmune tests revealed a positive antinuclear antibody (ANA); however, subsequent serology testing was negative for anti-dsDNA antibodies, antineutrophil cytoplasmic antibody (ANCA) antibodies, and rheumatoid factor (see Table 2). There were also no significant changes from the initial investigations conducted 10 days ago. Overall, the results were deemed unremarkable.

**Table 2:** Comprehensive autoimmune panel and blood culture evaluation on the follow-up visit 10 days later. The autoimmune workup shows positive ANA with subsequent tests revealing normal limits of anti-dsDNA, c-ANCA, p-ANCA, and rheumatoid factor. Blood cultures were also negative. ANCA: antineutrophil cytoplasmic antibody; p-ANCA: perinuclear ANCA; c-ANCA: cytoplasmic ANCA; ELISA: enzyme-linked immunosorbent assay

Category	Test	Result	Reference Range	Units
Autoimmune Tests	Anti-Nuclear Antibody (ANA)	Positive	Negative	–
	ANCA Antibody, c-ANCA	Negative	Negative	–
	ANCA Antibody, p-ANCA	Negative	Negative	–
	Anti-dsDNA (DNA Double-Stranded Ab, IgG, ELISA)	<10	<30	IU/mL
	Rheumatoid Factor (RF)	5.4	<14	IU/mL
Microbiology	Blood Cultures	Negative	Negative	–

**Figure 1:**
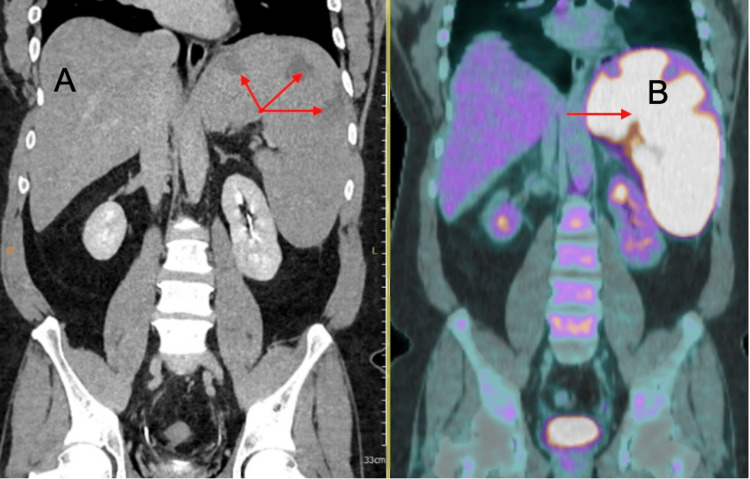
Coronal CT and PET scan A (left): Coronal CT image showing marked splenomegaly and areas of low attenuation consistent with splenic infarcts (red arrows). B (right): A fludeoxyglucose (FDG) PET/CT scan demonstrating increased FDG uptake in the spleen (red arrow), suggestive of lymphomatous involvement.

The physical examination findings and laboratory results indicated an underlying inflammatory process. Consequently, a repeat CT scan of the chest, abdomen, and pelvis (Figure 1A) was ordered to evaluate and confirm the cause of suspected splenomegaly. The scan revealed splenic enlargement of 19 cm, along with multiple wedge-shaped peripheral splenic infarcts that had not been observed previously.

Following these abnormal CT findings, a positron emission tomography (PET)/CT scan with fludeoxyglucose (FDG) (Figure 1B) was performed to rule out extra-pulmonary tuberculosis or an occult malignancy. As shown in Figure 1B, the hypermetabolic spleen was strongly suggestive of lymphomatous involvement. Subsequently, the PET/CT scan showed multiple small bilateral cervical lymph nodes, with the largest measuring 1.4 cm, as well as right axillary lymph nodes, the largest measuring 1 cm. It also revealed a diffusely hypermetabolic enlarged spleen measuring 19.3 cm, with a maximum standardized uptake value (SUVmax) of 16.7.

**Figure 2:**
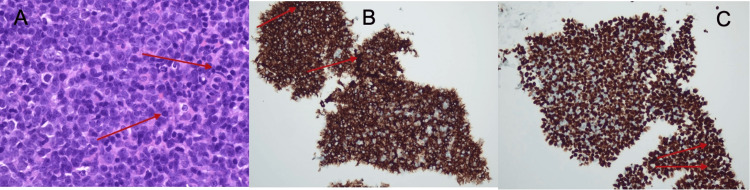
Histopathological and immunohistochemistry analysis of the right submandibular lymph node biopsy. A (left): Hematoxylin and Eosin stain at 60x magnification showing infiltration by large malignant lymphoid cells with pleomorphic nuclei and scant cytoplasm (red arrows). B (middle): CD20 immunostaining at 20x magnification demonstrating diffuse membranous positivity in neoplastic B cells (red arrows). C (right): Ki67 immunostaining at 20x magnification showing a high proliferative index (90%) in malignant cells (red arrows).

Following the increased FDG uptake in the spleen along with lymphomatous involvement, an ultrasound-guided percutaneous Tru-Cut biopsy of a superficial right submandibular lymph node was performed to identify the etiology of the lymphoadenopathy and spleenomegaly. The findings revealed infiltration by large, pleomorphic malignant lymphoid cells with scant cytoplasm. Immuno-histochemistry showed neoplastic cells expressing CD 20, CD79a, MUM1, BCL2, BCL6, and CMYC with a Ki67 proliferative index of 90% (Figure 2A, 2B, 2C). CD10 was negative. A final diagnosis of DLBCL, a non-germinal centre B-cell (non-GCB) subtype with dual expression of BCL2 and CMYC, was made. A bone marrow biopsy was deferred as the patient had early-stage disease (Ann Arbor stage II) based on imaging, and the findings were not expected to alter the planned first-line management.

**Figure 3:**
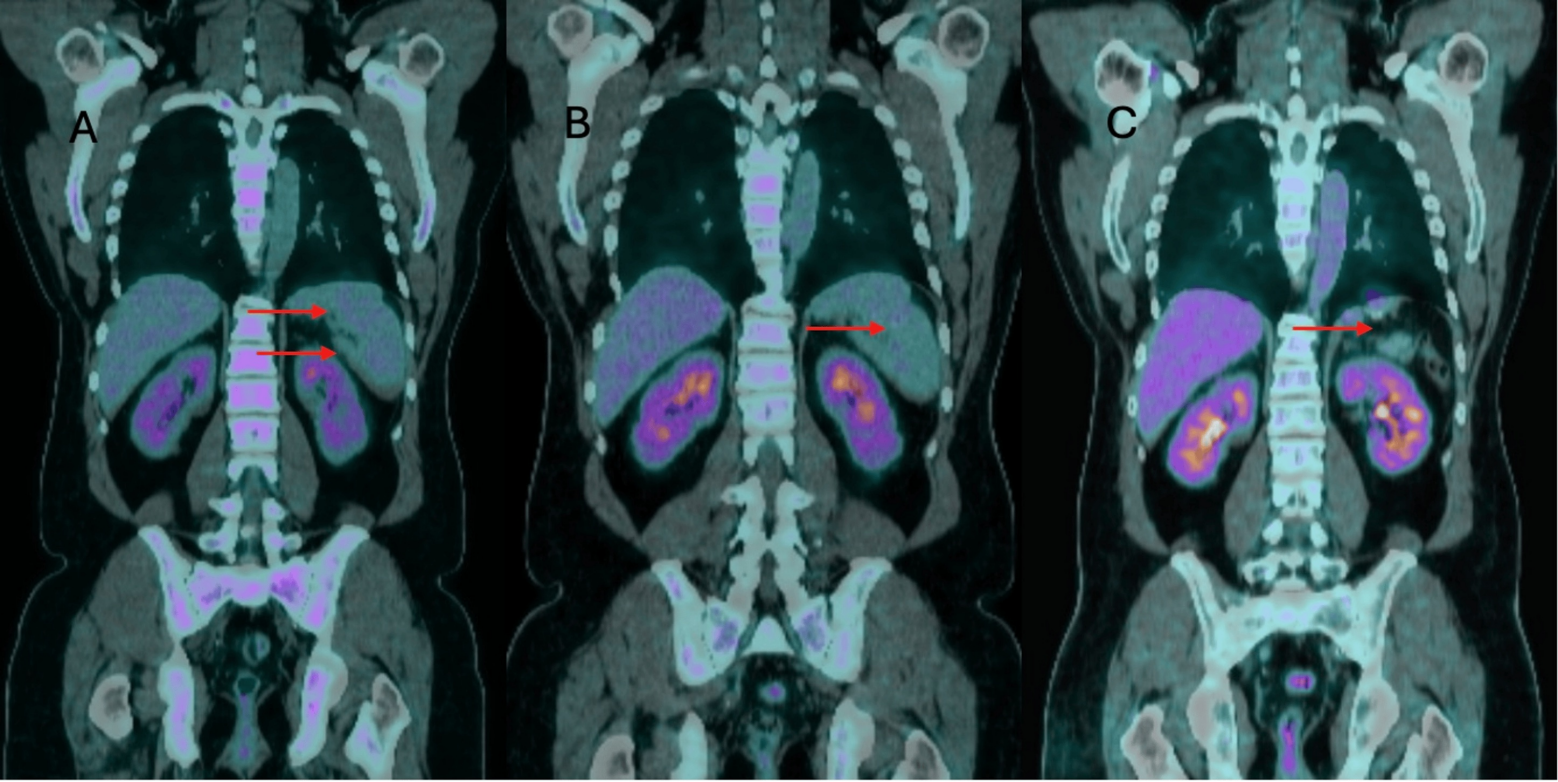
FDG PET/CT response assessment after cycle 4 and 6 after chemo-immunotherapy followed by metabolic resolution post-splenectomy. A (left): PET/CT following cycle 4 of chemo-immunotherapy showing partial metabolic response with residual uptake in enlarged spleen (red arrows). B (middle): PET/CT after completion of cycle 6 demonstrating further reduction in splenic metabolic activity and volume with persistent fludeoxyglucose (FDG) uptake (red arrow). C (right): PET/CT showing complete metabolic response post-splenectomy (red arrow indicates the anatomical site of removed spleen).

The patient was commenced on chemo-immunotherapy, initially with the R-CHOEP regimen (rituximab, cyclophosphamide, doxorubicin, vincristine, prednisolone, and etoposide), which was later modified to the standard R-CHOP (rituximab, cyclophosphamide, hydroxydaunorubicin, vincristine, and prednisone) therapy due to emergent grade 3 neutropenia without sepsis. A reassessment PET/CT scan after cycle 4 showed a partial response with focal residual FDG uptake in the spleen, which reduced in metabolic volume but persisted after cycle 6 of chemo-immunotherapy, as shown in Figure 3A, 3B. Infarcted areas in the spleen were constant from before chemotherapy.

An elective splenectomy was performed as a therapeutic measure for possible residual lymphoma. Spleen histopathology, however, did not demonstrate residual lymphoma, and a follow-up PET/CT scan six weeks later showed complete metabolic remission, which is maintained, as shown in Figure 3C.

In summary, the initial investigations revealed a mildly elevated monocyte count, which could be an early hematologic indicator of a potential myeloproliferative disorder or monocyte-associated lymphoma variant. The patient's elevated inflammatory markers, along with a positive ANA test, might have suggested an autoimmune cause for his condition; however, further autoimmune testing returned unremarkable results. Imaging studies showed significant splenomegaly with areas of low attenuation indicative of infarction, along with increased FDG uptake that suggested lymphomatous infiltration. Histopathological evaluation confirmed the diagnosis of diffuse large B-cell lymphoma, characterized by the infiltration of large atypical lymphoid cells that tested positive for CD20 and exhibited a high Ki-67 proliferative index. Serial PET/CT scans documented a metabolic improvement, progressing from a partial response after four cycles of chemo-immunotherapy to a complete metabolic remission following splenectomy.

## Discussion

The presentation of DLBCL is highly variable, with most individuals developing palpable diffuse lymphadenopathy and about a third presenting with extra nodal manifestations, such as hepatosplenomegaly [3].

While painless splenomegaly has widely accepted differential diagnoses, acute symptomatic splenic infarcts are an uncommon clinical problem. It has been described to occur in sickle cell disease, infective endocarditis, systemic vasculitis and acute infections causing rapid splenic enlargement (e.g. acute infectious mononucleosis and acute brucellosis). Hematologic malignancies can also rarely present with catastrophic splenic complications, including spontaneous rupture, underscoring the organ’s vulnerability in these diseases [4]. Extra-pulmonary tuberculous involvement of the spleen was also considered in this patient, given his history of childhood tuberculosis; however, the acute onset of symptoms made this diagnosis less likely [5].

In this case, the initial presentation of chest pain and fever without lymphadenopathy posed a diagnostic challenge. The chest pain was later localized to the left upper quadrant, corresponding to splenic pathology. This highlights an important red flag, where the sudden onset of upper abdominal pain in the absence of infection should raise suspicion for splenic infarction and potential hematologic malignancy.

A PET/CT scan was invaluable in identifying an alternative site for a diagnostic biopsy, given the significant risk of haemorrhage from a splenic biopsy. As widely recognised, FDG PET/CT also upstages DLBCL in 5-15% of cases [6] and showed extranodal (splenic) involvement in this patient.

Isolated splenomegaly is seen in fewer than 1% of non-Hodgkin B-cell lymphomas [3]. Painful splenic infarcts due to splenic involvement with DLBCL have only been reported rarely in the literature [7,8]. In comparable cases described by Kanani and Sheikh (2024) [4], lymphomatous infiltration resulted in vascular occlusion or parenchymal compression, predisposing to ischemia and infarction. We similarly postulate that the rapid, expansive growth of the lymphomatous tissue within the spleen outstripped its blood supply, leading to ischemia and subsequent infarction.

The histopathologic subtype of DLBCL in this patient is the non-GCB subtype with MYC/BCL-2 dual expression and a high Ki-67 proliferative index, consistent with a biologically proliferative tumour, which is reflected in the clinical presentation of acute splenomegaly with splenic infarcts.

In summary, progressive spleenomegaly with infarction, even in the absence of infection and lymphadenopathy, should prompt further investigation for hematologic malignancies such as DLBCL. Early recognition of this association is crucial to guide timely management. 

## Conclusions

Isolated, painful splenic infarcts require a thorough diagnostic evaluation to rule out common conditions such as infective endocarditis, autoimmune vasculitis, and other infectious causes. This case highlights that isolated painful splenic infarcts can be an early indication of aggressive lymphoma, such as DLBCL. It emphasizes the crucial role of PET/CT scans in guiding biopsies and the necessity of a detailed histopathological examination to confirm the diagnosis. Additionally, it is important to educate both general practitioners and specialists about the significance of key pathological features and genetic subtypes. For instance, a high Ki-67 index and MYC/BCL2 expression are associated with worse outcomes. Understanding these factors is essential for early identification and timely management of the condition.
